# Genetic spectrum and genotype–phenotype correlations in *DNAH5*-mutated primary ciliary dyskinesia: a systematic review

**DOI:** 10.1186/s13023-025-03596-5

**Published:** 2025-03-03

**Authors:** Meihua Dong, Xu Shi, Yawen Zhou, Jielin Duan, Li He, Xiaonan Song, Zhiwen Huang, Ruchong Chen, Jing Li, Nan Jia

**Affiliations:** Department of Allergy and Clinical Immunology, National Clinical Research Center for Respiratory Disease, State Key Laboratory of Respiratory Disease, Guangzhou Institute of Respiratory Health, the First Affiliated Hospital of Guangzhou Medical University, Guangzhou Medical University, Guangzhou, Guangdong China

**Keywords:** Primary ciliary dyskinesia, *DNAH5*, Genotype–phenotype correlation

## Abstract

**Background:**

Primary ciliary dyskinesia (PCD), a rare ciliopathy disorder, is caused by variants in multiple genes, with *DNAH5* being one of the most frequently implicated. However, the precise relationship between variant type or location in the *DNAH5* gene and the clinical heterogeneity remains elusive. The present systematic review aims to provide critical insights into the impact of the molecular nature of *DNAH5* variants on PCD phenotypes.

**Methods:**

We enrolled all reported cases of PCD with biallelic pathogenic variants in the *DNAH5* gene to date, and evaluated genotype–phenotype correlations in these patients, employing truncating (TV) and missense (MV) variant-carrying as grouping criteria.

**Results:**

A total of 323 PCD patients with the *DNAH5* variants were included, with 14.55% of these patients were diagnosed as Kartagener syndrome. Pediatric and adult patients exhibited distinct clinical features, including varying incidences of bronchiectasis, infertility, neonatal respiratory distress (NRD), ciliary ultrastructural defects distributions, and lung function (all *p* < 0.05). With regard to mutational patterns, truncating variants in *DNAH5* were clustered in the 1200–3200 amino acid region, and were more prevalent in children compared to adult (*p* < 0.0001). Most missense variants are clustering in the linker, AAA + ATPase and AAA-lid domains. The most frequently observed mutation, c.10815delT, was prevalent in Europe and America, whereas c.8030G > A was more common in China and Asia. In terms of genotype–phenotype correlations, individuals with the TV/TV genotype exhibited a higher proportion of NRD and earlier onset compared to those with MV-carrying genotypes, both in overall population and in pediatric patients (all *p* < 0.05). Patients with the TV/TV genotype exhibited worse lung function compared to those with MV-carrying genotypes.

**Conclusion:**

The study underscores the broad mutational spectrum and high phenotypic heterogenicity in *DNAH5*-related PCD patients. The presence of biallelic truncating variants may predispose patients to earlier disease onset and poorer lung function.

**Supplementary Information:**

The online version contains supplementary material available at 10.1186/s13023-025-03596-5.

## Introduction

Primary ciliary dyskinesia (PCD) is a set of rare inherited diseases, with an estimated global incidence of approximately 1 in 7500 individuals [1]. PCD arises due to genetic variants that lead to structural and functional defects in the motile cilia, subsequently causing dysfunction in corresponding tissues or organs and giving rise to a series of clinical manifestations [2, 3]. The symptoms of PCD typically include chronic infections affecting the upper and lower airways or ears, laterality defects, subfertility, and, in rare instances, hydrocephalus. Notably, approximately 50% of PCD cases are diagnosed as Kartagener syndrome (KS), a specific subtype characterized by a triad of chronic sinusitis, bronchiectasis, and situs inversus. Given the high genetic and clinical heterogenicities, there is no singular “Gold standard” test available for PCD diagnosis. Two leading guidelines recommend diagnostic strategies that incorporate a combination of clinical features, nasal nitric oxide (nNO) measurements, transmission electron microscopy (TEM) for ciliary structure analysis, and genetic testing [4, 5]. Other methods for detecting ciliary function, such as high-speed video analysis (HSVA) and immunofluorescence analysis (IF), have demonstrated their utility but are not yet widely used in clinical diagnosis.

Genetic studies have identified more than 50 ciliopathy-associated genes as PCD causative genes [1, 6]. The majority of PCD patients inherit the condition in an autosomal recessive manner, but exceptions include *FOXJ1*-PCD (which is autosomal dominant) and *PIH1D3*-PCD and *OFD1*-PCD (which are X-linked) [7–12]. One of the most frequently implicated gene is *DNAH5* (MIM ^#^603335), accounting for 15–29% of PCD patients in North America and Europe, and 18% in China [13–17]. The *DNAH5* gene is located on chromosome 5p15.2 and encodes a protein of 4624 amino acids (aa). This protein functions as the heavy chain subunit of the axonemal outer dynein arm (ODA), which, along with other axonemal proteins, forms the ODAs. ODAs and inner dynein arms (IDAs) are essential components of motile cilia, enabling ciliary movement in an ATP-dependent manner [7, 18, 19]. DNAH5 comprises several functional domains. An N-terminal cargo-binding domain which interacts with the intermediate and light chains of ODA, while the core motor domain consists of a hexameric AAA + ATPase (AAA1–AAA6) responsible for ATP-dependent enzymatic activity. A stalk domain harbors an ATP-sensitive microtubule-binding site (MTBD) crucial for microtubule interactions. Additionally, a linker domain and a C-terminal motor domain are present [7, 20–22]. The linker domain undergoes conformational changes in response to the absence or presence of ATP, triggering the stalk coiled-coil regions (CC1 and CC2) sliding against each other. This sliding motion, coupled with the MTBD’s ability to strongly bind or detach from microtubules, generates the force necessary for ciliary movement [23, 24].

Over the past decades, numerous genotype–phenotype correlation analyses have been conducted, offering valuable insights into understanding the impact of genetic variations on ciliary function and clinical manifestations. Notably, genetic associations with disease severity have been explored in PCD patients. Variants in the *CCDC39* or *CCDC40* genes are more likely associated with worsened lung function, reduced growth indices, and a higher incidence of infertility [23, 25]. Conversely, variants in the *DNAH11* [24], *DNAH9* [26], and *RSPH1* [27] genes tend to manifest in milder respiratory symptoms. Interestingly, genetic variants in the *DNAH5* gene exhibit variable effects on lung function, underscoring the complexity of genotype–phenotype relationships in PCD [28]. Previous reports have demonstrated that nonsense variants in the *DNAH5* gene lead to a complete absence of ODA in respiratory cilia. However, a splice site variant predicting the loss of exon 75 does not result in total ODA absence [29], indicating that phenotypic variability in PCD may be influenced by the nature and location of variants within *DNAH5*. Nevertheless, large-scale studies are necessary to validate this hypothesis. In this manuscript, we conducted a systematic review of 440 publications, ultimately including 323 patients from 66 studies reporting PCD patients with biallelic *DNAH5* mutations. Our aim was to investigate the correlation between the molecular characteristics of *DNAH5* variants and the phenotypic manifestations of PCD.

## Methods

### PRISMA guidelines

This system review analysis was performed following the Preferred Reporting Items for Systematic Reviews and Meta-Analyses (PRISMA) guidelines, as well as the practice guidelines outlined by Siddaway et al. [30]. Additionally, we utilized the PICOS framework (participants, interventions, comparators, outcomes, and study design) to establish eligibility criteria for including studies in the review (Table S1) [31].

### Search strategy

We conducted a comprehensive literature search across multiple databases, including PubMed, Scopus, ScienceDirect, as well as in the Chinese full-text databases such as Wanfang Data Knowledge Service Platform, CNKI, and China Science and Technology Journal Database. This extensive search was conducted without any language restrictions, targeting studies published between January 1984 and June 2023. The focus was specifically on studies describing patients with PCD who harbored variants in the *DNAH5* gene (NM_001369.3). To ensure the capture of all relevant information, we carefully crafted the search terms for each database, aiming for both brevity and inclusivity.

PubMed: ("Kartagener syndrome"[Title/Abstract] OR "primary ciliary dyskinesia"[Title/Abstract] OR "Polynesian bronchiectasis"[Title/Abstract] OR "immotile cilia syndromes"[Title/Abstract] OR "ciliary motility disorders"[MeSH Terms]) AND ("DNAH5"OR "dynein axonemal heavy chain 5" OR "Axonemal Dyneins"[MeSH Terms])).

Scopus: TITLE-ABS-KEY (("Kartagener syndrome" OR "primary ciliary dyskinesia" OR "Polynesian bronchiectasis" OR "immotile cilia syndromes" OR "ciliary motility disorders") AND ("DNAH5" OR "dynein axonemal heavy chain 5" OR "Axonemal Dyneins")).

Science Direct: (("Kartagener syndrome" OR "primary ciliary dyskinesia" OR "Polynesian bronchiectasis" OR "immotile cilia syndromes" OR "ciliary motility disorders") AND ("DNAH5" OR "dynein axonemal heavy chain 5" OR "Axonemal Dyneins")).

Wanfang Data Knowledge Service Platform: ALL: ((Kartagener OR cilia) AND (DNAH5)).

CNKI: TKA = (Kartagener OR cilia) AND TKA = (DNAH5).

China Science and Technology Journal Database: ALL = (Kartagener OR cilia) AND (DNAH5).

### Study selection

Three independent reviewers (M.D., X.S. & Y.Z.) with expertise in PCD assessed all studies for inclusion (Fig. S1). Following a full-text review, duplicate participants were identified and excluded based on a comprehensive analysis that considered factors such as mutations, clinical presentation, study origin, authorship, and reference list. Any discrepancies in the inclusion/exclusion decisions were resolved through discussion and consensus with a fourth investigator (N.J).

Inclusion criteria: 1) the studies with full text available; 2) the studies reported a genetic diagnosis with the biallelic pathogenic variants in the *DNAH5* gene. Pathogenic variants were classified as those predicted to result in loss-of-function protein products, including nonsense and frameshift mutations. Missense variants were evaluated for pathogenicity using computational prediction tools (PolyPhen-2, SIFT, Mutation Taster, and REVEL) and ClinVar database [32]; and 3) the studies met the diagnostic criteria for PCD. The diagnosis of PCD was established by the authors of each study, adhering to the diagnostic consensus prevalent at the time of the respective study. The diagnostic criteria encompassed the presence of classic symptoms, particularly KS, a subtype of PCD characterized by the triad of situs inversus, chronic sinusitis, and bronchiectasis; as well as abnormalities in ciliary ultrastructure or ciliary movement; and genetic analysis.

Exclusion criteria: 1) studies without peer review; 2) studies did not meet the diagnostic criteria; 3) studies lacking genetic information; 4) patients harboring heterozygous or hemizygous variants in *DNAH5*; and 5) patients with missense variants that did not fulfill the criteria for pathogenicity. Specifically, missense variants were excluded if: 1) at least two of the aforementioned algorithms predicted to be benign, tolerated, or neutral in effect; and 2) variants classified as benign, likely benign, or variant of uncertain significance, or those not reported in ClinVar (Table S2). Fourteen missense variants with uncertain pathogenicity were excluded from the analysis, including A376S, N549D, D556G, R683W, Q1398R, R1654Q, R1840W, E2143V, I2600M, R3000Q, V3784M, R4158W, R4358Q, and R4429Q.

### Data extraction

The data was extracted independently by two researchers (M.D. & Y.Z.) using a pre-prepared data extraction spreadsheet, and subsequently verified by a third independent reviewer (N.J.). Any discrepancies were resolved through discussion and consensus among all participants. The extracted information included: 1) patient characteristics, including origin, age at disease onset, age at disease diagnosis, and gender; 2) clinical characteristics, encompassing typical presentations, co-morbidities, and associated pathogens; 3) clinical and laboratory examination results, specifically chest CT scans, nNO measurements, pulmonary function tests, HSVA and TEM; and 4) molecular genetic data, including genotypes, variant types and their corresponding locations, as well as associated protein alterations. Detail information was listed in Table S3. *DNAH5* variants were named in accordance with the Human Genome Variation Society (HGVS) nomenclature, utilizing the NCBI transcript ID NM_001369.3 as the reference sequence. The nucleotide numbering of the coding sequences commenced with the A of the ATG translation initiation site, designated as nucleotide 1. Protein alterations were inferred from the corresponding genomic changes.

### Quality assessment and risk of bias

To assess the potential risk of bias within the included studies, we utilized the Joanna Briggs Institute (JBI) critical appraisal checklists specifically for case reports, case series, and diagnostic test accuracy studies [33–35]. Given the heterogeneous nature of the research designs, it was imperative to adopt multiple evaluation tools to ensure a comprehensive assessment of the literature. To further bolster the rigor and validate the appropriateness of the selected articles, an additional independent reviewer (X.S.) rigorously screened 10% of the full-text articles. Each article underwent a rigorous review process conducted by two independent reviewers (M.D. & Y.Z.). Only studies demonstrating moderate to high levels of methodological rigor and scientific quality were subsequently included in the category analysis and subsequent discussion (Table S2).

To account for potential confounding effects, the following variables were adjusted and normalized. Firstly, *DNAH5* variants were named using the NCBI transcript ID NM_001369.3 as a reference sequence to ensure consistency across studies. Secondly, we present nNO as either normal (< 77 nl/min or < 200 ppb) and abnormal, rather than specific values, to mitigate potential differences in measuring instruments. Thirdly, the ciliary beating pattern (CBP) was classified into three categories: normal or nearly normal cilia, dyskinetic cilia (impaired cilia with residual activity) and immotile cilia, to standardize the analysis.

### Statistical analysis

The data were analyzed using SPSS (version 26) and subsequently visualized with GraphPad Prism (version 9.0.0.121). Depending on suitability, the data were presented either as median (accompanied by the interquartile range) or as a ratio of n/N (indicating the frequency as a percentage). Categorical variables underwent comparison via the chi-squared test, whereas continuous variables were subject to either the Kruskal–Wallis test or the Mann–Whitney U test, depending on the context. To explore the correlation between genotype and clinical variables, linear models within the GraphPad Prism software were employed. All statistical analyses were performed in a two-sided manner, with a p-value less than 0.05 denoting statistical significance.

## Results

### Selection results

**Fig. 1:**
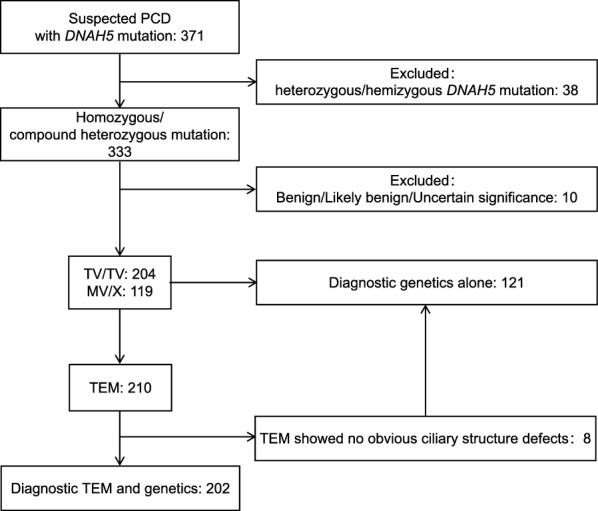
Diagrammatic overview of the participant enrollment flowchart. Abbreviations: PCD, primary ciliary dyskinesia; TEM, transmission electron microscopy; TV/TV, biallelic truncating variants; MV/X, carrying at least one missense variant

A total of 440 articles was included in our initial search, after duplicates were excluded. Subsequently, we excluded an additional 303 articles that were either not original studies, not topic-related, or focused on animal studies (Fig. S1). Six articles were further excluded due to the unavailability of the full text. After reading the full text of the remaining 131 studies, we excluded an additional 65 articles, including 20 with unrelated topics, 44 lacking genetic information, and 1 overlapping study. Finally, we included the remaining 66 studies reporting on 323 PCD patients with biallelic *DNAH5* mutations. Amont these patients, 202 were diagnosed by both TEM and genetic testing, while 121 were diagnosed solely by molecular genetics testing (Fig. 1). Three of the 66 patients had abnormal nNO values. Intriguingly, 47 patients were diagnosed as KS, accounting for only 14.55% of PCD cases with *DNAH5* mutations.

### Clinical features of PCD patients with *DNAH5* mutations

**Fig. 2:**
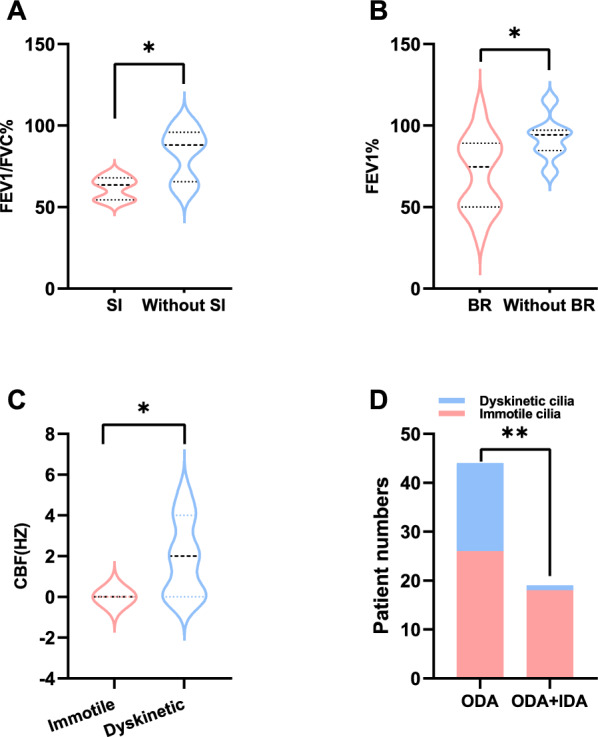
The clinical characteristics of PCD patients harboring *DNAH5* mutations: **A** The FEV1/FVC% between patients with (red) and without (blue) situs inversus. **B** The FEV1% between patients with (red) and without (blue) bronchiectasis. **C** The cilia beat frequency (CBF) between immotile cilia (red) and dyskinetic cilia (blue) subgroups. **D** The prevalence of immotile cilia (red) and dyskinetic cilia (blue) between patients with ODA defects and ODA + IDA defects. Abbreviations: SI, situs inversus; BR, bronchiectasis; FEV1%, forced expiratory flow in 1 s in predicted; FEV1/FVC%, forced expiratory flow in 1 s / forced vital capacity in predicted; ODA, outer dynein arm; IDA, inner dynein arm; Immotile cilia, immotile or loss of ciliary motion (red); Dyskinetic cilia, cilia with minimal residual, limited range, stiff or dyskinetic movements, or with low ciliary beat frequency (blue). Statistically significant differences are denoted with asterisks (*p < 0.05, **p < 0.01, ***p < 0.001, ****p < 0.0001)

Baseline demographic and clinical characteristics, ciliary ultrastructure and movements, and other indices were shown in Table 1. Out of 323 *DNAH5*-mutated PCD patients, 86 (51.19%) were male, and 58 (35.80%) were categorized as adult patients (age at diagnosis over 16 years old). Significant differences in incidences of neonatal respiratory distress (NRD) (66.29% vs. 39.02%, *p* = 0.0043), bronchiectasis (52.17% vs. 82.00%, *p* = 0.0009), and infertility (0.00% vs. 64.28%, *p* = 0.0275) were found between pediatric and adult groups. Adult patients exhibited significantly higher frequencies of ODA defects in cilia (91.89% vs. 68.95%, *p* = 0.0082). Conversely, pediatric patients showed a significantly higher occurrence of ODA + IDA defects (17.57% vs. 2.70%, *p* = 0.0323). Lung function assessments demonstrated significantly poorer performance in adult patients compared to pediatric counterparts (FEV1% 50.40 vs. 87.60, *p* = 0.0032, FEV1/FVC% 63.64 vs. 91.35, *p* = 0.0019, respectively). Compared to male, female PCD patients had a significantly higher prevalence of bronchiectasis (76.37% vs. 57.63%, *p* = 0.0340). Furthermore, patients with situs inversus exhibited significantly reduced FEV1/FVC% (61.66% vs 82.98%, *p* = 0.0152), while those with bronchiectasis had poorer FEV1% values (74.70 vs.94.30, *p* = 0.0329) (Fig. 2A and B).

**Table 1:** Clinical characteristics of PCD patients with variants in *DNAH5*

Parameters	Total (n = 323)	Pediatric (n = 104)	Adult (n = 58)	*P* value	Female (n = 82)	Male (n = 86)	*P* value
Demographic							
Male^a^	86/168(51.19%)	44/82(53.66%)	25/43(58.14%)	0.6336	0/82(0.00%)	86/86(100.00%)	1.0000
Adult^a^	58/162(35.80%)	0/104(0.00%)	58/58(100.00%)	1.0000	18/56(32.14%)	25/69(36.23%)	0.7065
Age at onset (years)^*^	0.0025(0, 1)	0(0, 0.3)	0.8(0, 9.8)	0.0673	0.0025(0, 5)	0.0833(0, 0.4)	0.7613
Age at diagnosis (years)^*^	11.8(7, 19)	8(4.3, 11)	27(18, 35)	** < 0.0001**	11.6(8, 19)	11.7(6, 18)	0.2734
Clinical manifestations							
Infections^a^	15/22(68.18%)	8/13(61.54%)	6/8(75.00%)	0.6557	7/11(63.64%)	8/11(72.73%)	1.0000
Pseudomonas aeruginosa^a^	7/15(46.67%)	2/8(25.00%)	5/6(83.34%)	0.0987	3/7(42.86%)	4/8(50.00%)	1.0000
Other pathogen^a^	8/15(53.34%)	6/8(75.00%)	1/6(16.67%)	0.4231	4/7(57.14%)	4/8(50.00%)	1.0000
Neonatal distress^a^	86/143(60.14%)	59/89(66.29%)	16/41(39.02%)	**0.0043**	26/48(54.17%)	36/63(57.14%)	0.8475
Situs inversus^a^	112/187(60.22%)	57/104(54.81%)	32/56(57.14%)	0.7774	26/48(54.17%)	46/79(58.22%)	0.8347
Bronchiectasis^a^	92/139(66.19%)	36/69(52.17%)	41/50(82.00%)	**0.0009**	42/55(76.37%)	34/59(57.63%)	0.0340
Sinusitis^a^	140/162(86.42%)	78/91(85.71%)	42/49(85.71%)	1.0000	48/61(78.69%)	59/66(89.39%)	0.0993
Otitis media^a^	87/135(64.44%)	61/85(71.76%)	25/43(58.13%)	0.0854	27/48(56.25%)	31/56(55.36%)	1.0000
Infertility^a^	11/25(44.00%)	0/4(0.00%)	9/14(64.28%)	**0.0275**	2/10(20.00%)	9/15(60.00%)	0.1181
Cilia ultrastructure and movement							
Normal ultrastructure^a^	8/210(3.81%)	6/74(8.11%)	1/37(2.70%)	0.4208	4/48(8.33%)	4/55(7.27%)	1.0000
ODA defects^a^	165/210(78.57%)	51/74(68.92%)	34/37(91.89%)	**0.0082**	33/48(68.75%)	38/55(69.09%)	1.0000
ODA + IDA defect^a^	30/210(14.29%)	13/74(17.57%)	1/37(2.70%)	**0.0323**	7/48(14.58%)	10/55(18.18%)	0.7912
Other defects^a^	7/210(3.33%)	4/74(5.41%)	1/37(2.70%)	0.6631	4/48(8.33%)	3/55(5.45%)	0.8519
Immotile cilia^a^	58/83(69.88%)	25/39(64.10%)	9/17(52.94%)	0.5540	17/28(60.7%)	22/30(73.3%)	0.4035
Dyskinetic cilia^a^	25/83(30.12%)	14/39(35.90%)	8/17(47.06%)	0.5540	11/28(39.3%)	8/30(26.7%)	0.4035
Other indices							
FEV1%^*^	83.2(55.0, 93.7)	87.6(81.4, 96)	50.4(46.4, 76.4)	**0.0032**	69.3(50.2, 95.2)	85.4(81.1, 93.3)	0.3522
FVC%^*^	79.7(62.9, 105.3)	80.1(63.9,105.2)	79.3(43.5, 106.6)	0.5523	75(62.8, 87.6)	95.3(69.1, 108.2)	0.3050
FEV1/FVC%^*^	68.7(62.5, 90.8)	91.4(87.2,100.1)	63.6(55.9, 67.1)	**0.0019**	65.5(60.2, 89.7)	77.3(63.0, 94.9)	0.5185

Data expressed as ^*^median (interquartile range) or ^a^n/N (percentage). N is the total number of patients with available data

Pediatric participants were diagnosed with the disease at an age ≤ 16 years; Adult participants were diagnosed with the disease at an age > 16 years

TEM, transmission electron microscopy; IDA, inner dynein arm; ODA, outer dynein arm; Other defects, defects in radial spoke, central pair, micro-tubular disorganization, nexin bridges or no cilia detectable; Immotile cilia, immotile or loss of ciliary motion; Dyskinetic cilia, cilia with minimal residual, limited range, stiff or dyskinetic movements, or with low ciliary beat frequency (CBF); FEV1%, forced expiratory flow in 1 s in predicted; FVC%, forced vital capacity in predicted.

*P* value was calculated from the x^2^, Kruskal–Wallis or Mann–Whitney U test. Statistically significant value (*p* < 0.05) is marked in bold

In terms of ciliary ultrastructural abnormalities, 165 (78.57%) of 210 *DNAH5*-mutated PCD patients exhibited ODA defects, 30 (14.29%) had ODA + IDA defects, and 7 (3.33%) presented with other ciliary structural abnormalities, including radial spoke, central pair and microtubular disorganization defects. Notably, 8 patients (3.81%) displayed no obvious ciliary structure defects (Table S3). Analysis of CBF among 83 patients who underwent HSVA revealed 58 (69.88%) and 25 (30.12%) had immotile and dyskinetic cilia, respectively. CBF was significantly lower in patients with immotile cilia than those with dyskinetic cilia (*p* = 0.0283) (Fig. 2C). Compared to those with ODA defects, patients with ODA + IDA defects are more likely have immotile cilia (94.74% vs. 59.09%, *p* = 0.0060) (Fig. 2D).

### Genetic spectrum of *DNAH5*-mutated PCD patients

**Fig. 3:**
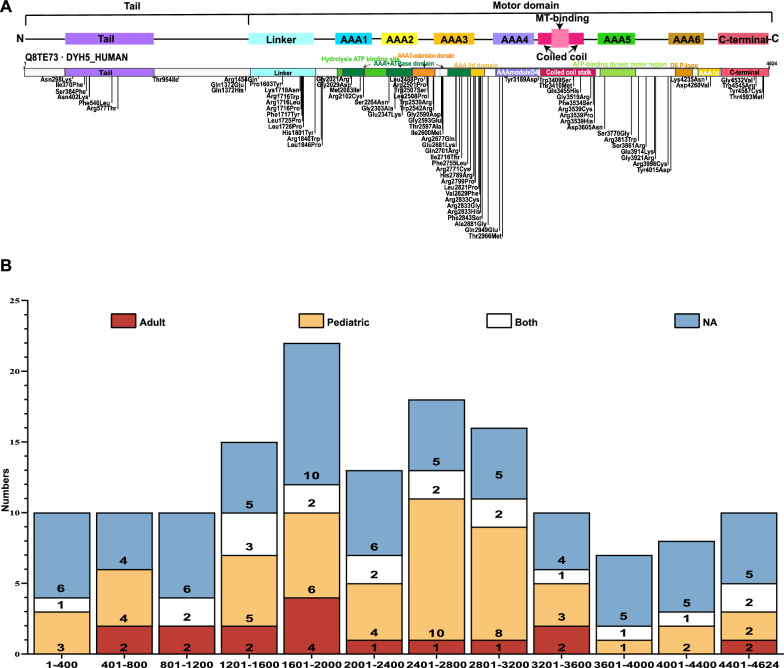
The distribution of *DNAH5* variants in PCD patients exhibits distinct patterns. **A** Missense variants are scattered across the DNAH5 protein, with various domains predicted by UniProt [58] and InterPro [59] databases highlighted in colored blocks. The upper model is an adaptation of King SM’s work [18], while the lower model is derived from InterPro database. The MT-binding region, representing the microtubule binding domain (MTBD). **B** In the distribution of truncating variants, variants have been categorized into intervals of 400 amino acids, and each column represents the count of variants within a specific group. Specifically, white columns denote variants exclusively found in adult PCD patients, black columns represent variants unique to pediatric cases, dark grey columns indicate variants identified in both pediatric and adult patients, and light grey columns with diagonal stripes signify patients with an unknown age of disease onset

Among the 323 patients harboring *DNAH5* mutations, 74 were homozygous and 249 were compound heterozygous genotype. A total of 299 pathogenic variants were identified, including 79 missense mutations, 5 in-frame insertions/deletions, and 215 protein-truncating genetic variants resulting from stop gains (91), frameshifts (63), large deletion (9), duplication (3) or splice variants (49) (Table S3). As shown in Fig. 3A, a significant proportion of missense variants were localized within specific domains, accounting for 61/78 (78.21%) mutations. Notably, 55 of these variants were particularly clustered in the motor domain, highlighting its crucial roles in DNAH5 functionality. Furthermore, the particularly high concentrated regions of missense variants were identified within linker domain specifically spanning 1403–1807 aa (21/323, 6.50%), the AAA + ATPase domain at 2582–2780 aa, and the AAA lid domain at 2770–2855 aa (27/323, 8.36%). The truncating variants were distributed across the DNAH5 protein, with a cluster in region spanning 1200–3200 aa, accounting for 56.38% of patients with truncating mutations. This region corresponds to the linker and AAA + domains, as illustrated in Fig. 3B. Truncating variants were more prevalent in pediatric patients compared to adults (44.96% vs. 23.49%, *p* < 0.0001). However, no difference in variant distribution was observed between pediatric and adult patients.

Importantly, no obvious correlation was established between specific variants and ciliary structural damage. A total of 143 variants were associated with exclusive ODA defects, distributed across the entire protein sequence. Forty-one variants could potentially cause both ODA and IDA defects, with 21 of these variants exclusively linked to ODA + IDA defects and 20 variants potentially associated with other ciliary ultrastructure defects (Fig. S2).

### Recurring pathogenic genetic variants

Thirty-four pathogenic variants were identified in three or more patients/families, suggestive of variant hotspots or founder variants (Table 2). Four variants were found in over 10 patients worldwide, including c.10815delT (31/280, 11.07%), 13458_13459insT (14/280, 5.00%), 13486C > T (12/280, 4.29%), and c.5563_5564insA (10/280, 3.57%). Six variants occurred in three or more patients in Asia, exhibiting higher frequencies compared to European and American populations. Specifically, c.5563_5564insA (located proximate to the linker domain) was the most frequent variant in Asia (6/72, 8.33%), whereas c.8030G > A (located within AAA3 domain) was the most common variant in China (4/48, 8.33%). Intriguingly, the variants c.8030G > A and c.6647delA, both focused with the Hydrolysis ATP binding site domain, were exclusively identified in the Chinese population, indicating their potential status as founder variants specific to China. Furthermore, it is noteworthy that the variants c.4348C > T, c.10384C > T and c.5281C > T were solely observed in patients of European and American ancestry, potentially indicating their emergence from founder effects. Within which, c.5281C > T, along with the other 14 C > T substitutions, resulted from variants at CpG sites, potentially indicating mutational hotspots [36]. On the other hand, c.4348C > T, c.10384C > T and c.3484C > T are variants generated at CpA sites.

**Table 2:** Recurring pathogenic variants in the *DNAH5* gene

Genomic mutation	Protein change (predicted)	Total (N = 280)	Euro-America (N = 125)	Asia (N = 72)	China (N = 48)	Unknown (N = 83)
c.670C > T^a^	p.Arg224*	3/280(1.07%)	1/125(0.80%)	NA	NA	2/83(2.41%)
c.832delG	p.Ala278Argfs*27	4/280(1.43%)	4/125(3.20%)	NA	NA	NA
c.1089 + 1G > A	p.Thr326-Pro363del or Thr326Valfs*25	3/280(1.07%)	3/125(2.40%)	NA	NA	NA
c.3037_3040delAGCG	p.Val1014Leufs*20	3/280(1.07%)	2/125(1.60%)	NA	NA	1/83(1.20%)
c.3484C > T^b^	p.Gln1162*	3/280(1.07%)	2/125(1.60%)	NA	NA	1/83(1.20%)
c.3905delT	p.Leu1302Argfs*19	3/280(1.07%)	2/125(1.60%)	NA	NA	1/83(1.20%)
c.4348C > T^b^	p.Gln1450*	6/280(2.14%)	6/125(4.80%)	NA	NA	NA
**c.5177 T > C**	p.Leu1726Pro	3/280(1.07%)	2/125(1.60%)	NA	NA	1/83(1.20%)
c.5281C > T^a^	p.Arg1761*	3/280(1.07%)	3/125(2.40%)	NA	NA	NA
c.5563_5564insA	p.Ile1855Asnfs*6	10/280(3.57%)	4/125(3.20%)	6/72(8.33%)	3/48(6.25%)	NA
c.5710-2A > G	p.Cys1904-Lys1909del	4/280(1.43%)	4/125(3.20%)	NA	NA	NA
c.5983C > T^a^	p.Arg1995*	4/280(1.43%)	2/125(1.60%)	2/72(2.78%)	NA	NA
**c.6249G > A**	p.Met2083Ile	4/280(1.43%)	2/125(1.60%)	NA	NA	2/83(2.41%)
**c.6304C > T**^a^	p.Arg2102Cys	3/280(1.07%)	NA	2/72(2.78%)	2/48(4.17%)	1/83(1.20%)
c.6647de1A	p.Lys2216Argfs*20	3/280(1.07%)	NA	3/72(4.17%)	3/48(6.25%)	NA
**c.6791G > A**	p.Ser2264Asn	3/280(1.07%)	2/125(1.60%)	NA	NA	1/83(1.20%)
**c.7502G > C**	p.Arg2501Pro	5/280(1.79%)	4/125(3.20%)	NA	NA	1/83(1.20%)
c.7915C > T	p.Arg2639*	4/280(1.43%)	3/125(2.40%)	1/72(1.39%)	1/48(2.08%)	NA
c.8029C > T^a^	p.Arg2677*	7/280(2.50%)	4/125(3.20%)	2/72(2.78%)	2/48(4.17%)	1/83(1.20%)
**c.8030G > A**	p.Arg2677Gln	4/280(1.43%)	NA	4/72(5.56%)	4/48(8.33%)	NA
**c.8311C > T**^a^	p.Arg2771Cys	3/280(1.07%)	NA	1/72(1.39%)	1/48(2.08%)	2/83(2.41%)
c.8383C > T^a^	p.Arg2795*	7/280(2.50%)	1/125(0.80%)	4/72(5.56%)	2/48(4.17%)	2/83(2.41%)
c.8440_8447del GAACCAAA	p.Glu2814fs*1	5/280(1.79%)	4/125(3.20%)	NA	NA	1/83(1.20%)
**c.8498G > A**	p.Arg2833His	4/280(1.43%)	2/125(1.60%)	1/72(1.39%)	1/48(2.08%)	1/83(1.20%)
c.9286C > T^a^	p.Arg3096*	3/280(1.07%)	1/125(0.80%)	2/72(2.78%)	1/48(2.08%)	NA
c.9502C > T^a^	p.Arg3168*	3/280(1.07%)	NA	3/72(4.17%)	2/48(4.17%)	NA
c.10384C > T^b^	p.Gln3462*	5/280(1.79%)	5/125(4.00%)	NA	NA	NA
**c.10615C > T**^a^	p.Arg3539Cys	3/280(1.07%)	2/125(1.60%)	NA	NA	1/83(1.20%)
**c.10616G > A**	p.Arg3539His	6/280(2.14%)	2/125(1.60%)	3/72(4.17%)	2/48(4.17%)	1/83(1.20%)
c.10815delT	p.Pro3606Hisfs*23	31/280(11.07%)	21/125(16.80%)	NA	NA	10/83(12.05%)
c.13194_13197delCAGA	p.Asp4398Glufs*16	5/280(1.79%)	5/125(4.00%)	NA	NA	NA
c.13285C > T^a^	p.Arg4429*	3/280(1.07%)	1/125(0.80%)	NA	NA	2/83(2.41%)
c.13458_13459insT	p.Asn4487fs*1	14/280(5.00%)	7/125(5.60%)	1/72(1.39%)	NA	6/83(7.23%)

Recurring pathogenic variants were defined as variants occurred in three or more patients / families. Euro-America, Patients of European and American ancestry; Unknown, Patients with unreported ancestry; NA: not available

^a^CpG site; ^b^CpA site. Missense variants presented in bold

### Genotype–phenotype correlations in PCD patients with *DNAH5* mutations.

**Fig. 4:**
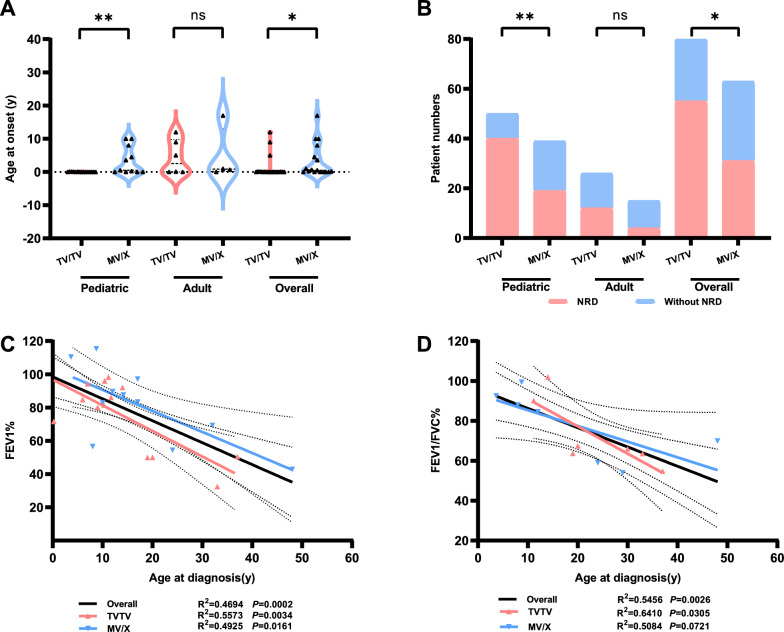
Exploring genotype–phenotype correlations in PCD patients harboring *DNAH5* variants reveal intriguing patterns. **A** The relationships between age of onset and genotypes (TV/TV versus MV/X) among pediatric, adult, and the overall patient population. **B** The bar chart highlights the number of patients experiencing neonatal respiratory distress (NRD; red bar) and those who did not show NRD (blue bar), segmented by different genotypes (TV/TV versus MV/X) across pediatric, adult, and the total cohort. **C, D** Scatter plots illustrate the correlation between FEV1% (C) and FEV1/FVC% (D) with age at diagnosis, segmented into the overall population (black), TV/TV genotype (red), and MV/X genotype (blue). Statistically significant differences are denoted with asterisks (*p < 0.05, **p < 0.01). TV/TV represents biallelic truncating variants, and MV/X stands for carrying at least one missense variant

We conducted a comprehensive analysis to investigate the correlations between clinical features and genetic profiles in *DNAH5*-mutated PCD patients, stratified based on their genotype: those harboring biallelic truncating variants (TV/TV), and those carrying at least one missense variant (MV/X). In pediatric patients, a significant difference was observed in the onset of disease symptoms between the TV/TV and MV/X genotypes. Patients possessing the TV/TV genotype displayed a significantly earlier onset of symptoms compared in the MV/X groups (0 month vs. 6 months, *p* = 0.0154). Furthermore, the prevalence of NRD was significantly higher in the TV/TV group, reaching 80.00% compared to 48.72% in the MV/X group (*p* = 0.0031). These findings were consistent with the trends observed in the overall population, confirming that the TV/TV genotype was associated with an earlier disease onset (0 months vs. 6 months, *p* = 0.0488) and a higher incidence of NRD (68.75% vs. 49.21%, *p* = 0.0250). However, in the adult subgroup, no statistically significant differences were observed (Fig. 4A, B). To address potential discrepancies arising from different age thresholds, we have defined age boundaries at 14 years for Fig. S5 and 18 years for Fig. S6 to ensure consistency. Pediatric patients with the TV/TV genotype exhibit a significantly earlier onset of symptoms compared to those in the MV/X groups, as demonstrated in Fig. S5C (*p* = 0.0205) and Fig. S6A (*p* = 0.0496). Additionally, the prevalence of NRD is significantly higher in the TV/TV group among pediatric patients, with statistical significance shown in Fig. S5D (*p* = 0.002) and Fig. S6B (*p* = 0.002). These trends are fully consistent with those observed when the age threshold is set at 16 years.

Both the overall population and patients with the TV/TV genotype exhibited a significant negative correlation between age at diagnosis and lung function parameters, specifically FEV1% and in FEV1/FVC% (all *p* < 0.05) (Fig. 4C-D). A similar negative correlation was also identified between age at diagnosis and FEV1% in patients harboring the MV/X genotype (*p* = 0.0161). Interestingly, the strength of these correlations appeared to be more robust in patients with the TV/TV genotype compared to those with the MV/X genotype, as evidenced by higher R^2^ values for FEV1% (R^2^ = 0.5573 vs. R^2^ = 0.4925) and FEV1/FVC% (R^2^ = 0.6410 vs. R^2^ = 0.5084), respectively. Moreover, the decline in lung function among individuals with the TV/TV genotype was observed to be more pronounced and accelerated with advancing age, as compared to the MV/X genotypes (Fig. 4C).

## Discussion

Understanding relationships between genetic defects and clinical consequences is crucial for better diagnosis and effective treatment strategies. However, due to the rarity and heterogeneity of rare diseases, it’s difficult to draw statistically significant conclusions. In such scenarios, systematic reviews emerge as a useful methodology for exploring genotype–phenotype correlations, as it allows for inclusion of all relevant studies and provides both quantitative and qualitative information [37]. Multiple systematic reviews and meta-analyses have been conducted, revealing associations between PCD genotypes with clinical manifestations, nNO and lung function indices, ultrastructural axonemal defects and ciliary beating frequency [38–41]. To date, large cohort studies or systematic reviews of genotype–phenotype correlations are focused on phenotypic diversity caused by different genes. With growing knowledge of clinical heterogeneity and pathophysiologic mechanisms, PCD is increasingly recognized as an umbrella term of motile ciliopathies with overlapping clinical features, rather than one single disease [42]. Consequently, there has been increasing attention paid to elucidating the relationships between variant nature and location in specific gene and phenotypic differences. In the present study, we provided the first comprehensive systematic review of genotype–phenotype associations in PCD patients harboring *DNAH5* variants (primary ciliary dyskinesia-3, CILD3, OMIM^#^608,644). Our findings confirmed the high phenotypic variability in *DNAH5*-mutated PCD patients and revealed significant phenotypic differences between pediatric and adult patients. Furthermore, we discovered that variant frequency or mutation hotspots exhibited geographical variation across different populations. Finally, our analyses suggested that patients carrying biallelic truncating variants may lead to earlier disease onset and worse lung function.

We found that the clinical manifestations of *DNAH5*-mutated PCD patients were highly variable, with randomization of LR asymmetry, respiratory symptoms and infertility, which is consistent with previous studies [28]. However, we do find significant phenotypic differences between pediatric and adult patients. Compared to adults, pediatric *DNAH5*-mutated PCD patients displayed a higher incidence of NRD but a lower occurrence of bronchiectasis and infertility. It is noteworthy that the higher incidence of NRD in pediatric patients might be attributed to earlier hospitalization earlier diagnosis. On the other hand, the higher incidence of bronchiectasis in adult patients could be the consequence of chronic respiratory infections. Although the age-related clinical variations may be influenced by retrospective data collection and less rigorous diagnostic confirmations, these results underscore the need for the development of separate management guidelines specifically for children and adolescent.

The severity of respiratory manifestations in PCD patients was primarily assessed by spirometric indices [39]. Our study revealed worse lung function among patients with situs inversus and bronchiectasis, with the latter finding consistent with previous literatures [43]. The prevalence of heterotaxy-related congenital heart disease was reported to be 200-folder higher in PCD patients compared to the general population [44]. An important consequence associated with congenital heart disease is the occurrence of complex cardiac defects and consequent decrease in pulmonary oxygenation, which could partially explain the decreased lung function in patients with situs inversus [45, 46]. It’s worth noting that ODA + IDA defects are more prone to lead to immotile cilia and a further decreased CBF in *DNAH5*-mutated PCD patients, which is consistent with the findings of a prospective study conducted in 2019 [47]. IDA damage has been reported to disrupt the normal waveform of cilia, diminishing both the beating angle and distance during each ciliary stroke [47, 48]. Given that CBF is generally believed to increase mucociliary transport [49], it’s reasonable to speculate that patients with ODA + IDA defects are more likely to exhibit poorer lung function compared to those with isolated ODA defects. Unfortunately, we are unable to find significant differences in lung function between these two groups in current study due to insufficient information. Further large cohort or long-term natural history studies are necessary to address the questions surrounding whether ODA + IDA defects lead to lower lung function and earlier disease presentation.

Previous researches have established that pathogenic *DNAH5* variants disturb the proper assembly of ODA complexes [16], and revealed that null dnah5 mice had absent ODA and normal IDA [50]. However, pathogenic *DNAH5* variants have also been reported resulting in ODA + IDA defects in clinical samples [51]. So, we further analyzed which *DNAH5* variants causes ODA + IDA defects, but we have failed to establish a clear connection between genotype and ciliary ultrastructure defect phenotype. One potential explanation for this observation is the possibility of false positive or negative results in TEM. There is secondary ciliary ultrastructural damage during sample processing and TEM detection. Studies had shown that 25–30% PCD patients initially diagnosed with IDA damage reverted to normal in repeat TEM tests [52]. Another possible reason is the existence of additional disease-causing PCD-associated genes responsible for IDA defects. As Pereira R, et al. (2019) reported a patient with ODA + IDA + nexin bridges defects who carried biallelic variants in both *DNAH5* and *DNAH7* (component of IDA) genes [51]. These variants led to reduced mRNA expression levels and abnormal ciliary subcellular location of both proteins [51]. Therefore, further research directions in PCD should include exploring the impact of multiple variants in different genes on phenotype, to gain a deeper understanding of the complex genetic factors that contribute to ciliary dysfunction and its associated clinical manifestations.

Several studies have reported potential genetic associations between disease severity and genotype in PCD patients. Individuals harboring biallelic pathogenic variants in *CCDC39*, *CCDC40* or *CCNO* genes, which can lead to microtubular defects or the absence of cilia, exhibited more severe disease manifestations, including earlier age at diagnosis, worse lung function and lower body mass indexes [28, 53]. Intriguingly, our research revealed for the first time that patients carrying biallelic truncating variants (TV/TV) in the *DNAH5* gene were associated with a higher prevalence of NRD, earlier disease onset, and faster deterioration of lung function (FEV1%) compared to those harboring missense variants (MV/X). This finding suggests a risk association between biallelic TV carriers and the severity of PCD, and highlights the potential influence of genotype on the rate of lung function deterioration over time, which is of significant clinical importance in understanding and managing age-related respiratory decline in at-risk populations. It is worth mentioning that, no significant difference in FEV1/FVC%, a crucial lung function parameter after lung size correction, was observed between the two genotypes. Moreover, due to limitations in sample size and missing data, we were also unable to carry out a FEV1 z-score analysis, which could have mitigated biases related to age, height, sex, or ethnic background [54]. It will be valuable to conduct longitudinal natural history or larger cohort in *DNAH5*-mutated PCD to confirm the association between TV/TV genotype and disease severity.

The postulated link between TVs and disease severity stems from the fact that TVs often lead to nonsense-mediated mRNA decay and subsequent loss of protein function [55]. We initially assumed that complete absence of *DNAH5* expression would lead to severe phenotype. However, immunofluorescence data revealed that variant DNAH5 proteins were indeed expressed but were mislocated in respiratory epithelial cells of PCD patients, regardless of mutational background [16, 56]. Patients carrying changes in N-terminal region of DNAH5 displayed a distinct accumulation of DNAH5 at the microtubule organizing centers, accompanied by its complete absence from the entire ciliary axonemes. Conversely, patients with C-terminal variants exhibited an absence of DNAH5 from distal ciliary axoneme (both DNAH5- and DNAH9-positive) but preserved in the proximal ciliary axoneme (DNAH5-positive and DNAH9-negative). The authors discovered a distinct pattern of DNAH5 mislocalization that is intimately linked to the frequency and characteristics of ciliary beating. Specially, N-terminal defects affecting both distal and proximal axoneme were associated with a significant reduction in cells exhibiting ciliary motility. However, this finding contrasts with our observation that over 40% of the variants responsible for immotile cilia located in C-terminal region of DNAH5. Furthermore, several variants causing immotile cilia are located within or adjacent to the tail domain of DNAH5, a region known to interact with intermediate chain of dynein [57]. It has been reported that outer-inner dynein (OID) linker, a complex of ODA intermediate chain 2 (IC2) and dynein regulatory complex 4 (DRC4), plays a crucial role in regulating both ODAs and IDAs, thereby controlling flagellar beating [57]. Thus, we speculated that changes in the C-terminal region of DNAH5 which disrupt normal formation of OID linker may also lead to decreased ODA and IDA activities, subsequently resulting in reduced ciliary beating. Further functional investigations are required to elucidate the specific mechanisms by which certain *DNAH5* variants affect ciliary structure and motility. Such insights will provide valuable information about the molecular pathology underlying PCD.

This study represents the largest investigation to date examining the genotype–phenotype correlation in *DNAH5*-mutated PCD patients, focusing on exploring the association between variant types (truncating and missense) / locations and highly diverse phenotypes. However, one of the major limitations of the current study is the incomplete data on crucial parameters, including lung function indices, TEM and CBF data. These omissions primarily stem from the reliance on published reports for data collection. Moreover, longitudinal follow-up data are essential to confirm the association between TV/TV genotype and decline rate of lung function. Finally, it is imperative to replicate these findings in a larger cohort of *DNAH5*-mutated PCD patients to gain a deeper understanding of the pathogenesis and progression of this disease.

## Conclusion

In the current study, we comprehensively examined the clinical manifestations and variant spectrum exhibited by patients with PCD harboring variants in *DNAH5*. Our findings reveal a remarkable heterogeneity in both the clinical presentations and the mutational landscape among these patients. Notably, individuals carrying biallelic truncating variants tend to manifest earlier disease onset and poorer lung function, thereby establishing a direct link between specific genotypes and the disease severity in *DNAH5*-mutated PCD patients. These observations underscore the crucial role of genetic diagnosis in prognosis of affected individuals and guiding timely intervention strategies.

## Supplementary Information

Additional file 1

Additional file 2

Additional file 3

Additional file 4

Additional file 5

Additional file 6

Additional file 7

Additional file 8

Additional file 9

Additional file 10

Additional file 11

Additional file 12

## Data Availability

All data relevant to the study are included in the article or uploaded as supplementary information.

## Abbreviations

PCD: Primary ciliary dyskinesia
TV: Truncating variants
MV: Missense variants
NRD: Neonatal respiratory distress
KS: Kartagener syndrome
nNO: Nasal nitric oxide
TEM: Transmission electron microscopy
HSVA: High-speed video analysis
IF: Immunofluorescence analysis
aa: Amino acids
ODA: Outer dynein arm
IDA: Inner dynein arm
PRISMA: Preferred reporting items for systematic reviews and meta-analyses
HGVS: Human genome variation society
JBI: Joanna briggs institute
CBP: Ciliary beating pattern
FEV1%: Forced expiratory flow in 1 s in predicted
FVC%: Forced vital capacity in predicted
OID: Outer-inner dynein
IC2: Intermediate chain 2
DRC4: Dynein regulatory complex 4
CBF: Ciliary beat frequency
MTBD: Microtubule binding domain
