# Supplementary material for: Genetic spectrum and genotype–phenotype correlations in DNAH5-mutated primary ciliary dyskinesia: a systematic review
Source: Orphanet J Rare Dis. 2025 Mar 3;20:97. doi: 10.1186/s13023-025-03596-5 (PMC11874857; doi:10.1186/s13023-025-03596-5)
Supplement: Supplementary file 1 — Additional file 1 [file 13023_2025_3596_MOESM1_ESM.docx]

**SUPPLEMENTARY FIGURE LEGENDS**

**Figure S1** Diagrammatic representation of the article enrollment flowchart.

**Figure S2** Distribution of *DNAH5* variants in PCD patients with ODA+IDA defects. Different domains were predicted by the Uniprot (https://www.uniprot.org/) and Interprot (https://www.ebi.ac.uk/interpro/) databases, and highlighted in colored blocks. Variants solely causing ODA+IDA defects were displayed beneath the DNAH5 protein sequence, whereas those potentially leading to additional conditions, including normal cilia, ODA defects or other defects, were shown above the protein sequence.

**Figure S3** Clinical features of PCD patients with *DNAH5* mutations after excluding studies of potentially low quality. (A)The FEV1/FVC% between patients with and without situs inversus. (B) The FEV1% between patients with and without bronchiectasis. (C) The cilia beat frequency (CBF) between immotile cilia and dyskinetic cilia subgroup. (D) The prevalence of immotile cilia between patients with ODA defects and ODA+IDA defects. SI, situs inversus; BR, bronchiectasis; FEV1%, forced expiratory flow in 1 second in predicted; FEV1/FVC%, forced expiratory flow in 1 second/ forced vital capacity in predicted; ODA, outer dynein arm; IDA, inner dynein arm; Immotile cilia, immotile or loss of ciliary motion; Dyskinetic cilia, cilia with minimal residual, limited range, stiff or dyskinetic movements, or with low ciliary beat frequency. Statistically significant differences are denoted with asterisks (**p* < 0.05, ***p* < 0.01, ****p* <0.001, *****p* < 0.0001).

**Figure S4** Genotype-phenotype correlations in PCD patients with *DNAH5* mutations after excluding studies with potential low quality. (A) Relationships of age at onset and genotypes (TV/TV vs MV/X) among children, adults and overall population. (B) Numbers of patients with (red bar) and without (blue bar) neonatal distress in patients with different genotypes (TV/TV vs MV/X) among children, adults and overall population. (C-D) The scatter plots of correlations between FEV1% (C) and FEV1/FVC% (D) with age at diagnosis in overall (black), TV/TV (red) and MV/X (blue) genotypes. **p* < 0.05, ***p* < 0.01. TV/TV, biallelic truncating variants; MV/X, carrying at least one missense variant..

**Figure S5** Clinical features and genotype-phenotype correlations in PCD patients with *DNAH5* mutations, with an adjusted cut-off age of 14 years at diagnosis. (A) The FEV1% between pediatric patients with and without neonatal respiratory distress. (B) The FVC% between pediatric patients with and without neonatal respiratory distress. (C) Correlation between age at onset and genotypes (TV/TV vs MV/X) among pediatric, adults and the overall population. (D) Numbers of patients with (red bar) and without (blue bar) neonatal distress in patients with different genotypes (TV/TV vs MV/X) within the pediatric, adults and the overall patient cohorts. **p* < 0.05, ***p* < 0.01. TV/TV, biallelic truncating variants; MV/X, carrying at least one missense variant.

**Figure S6** Clinical features and genotype-phenotype correlations in PCD patients with *DNAH5* mutations, with an adjusted cut-off age of 18 years at diagnosis. (A) Correlation between age at onset and genotypes (TV/TV vs MV/X) among pediatric, adults and the overall population. (B) Numbers of patients with (red bar) and without (blue bar) neonatal distress in patients with different genotypes (TV/TV vs MV/X) within the pediatric, adults and the overall patient cohorts. **p* < 0.05, ***p* < 0.01. TV/TV, biallelic truncating variants; MV/X, carrying at least one missense variant.

**Table S1** Eligibility criteria using PICOS framework (participants, interventions, comparators, outcomes, and study design).

**Table S2** The pathogenicity prediction of missense variants. To assess the pathogenicity of missense variants, we have employed several state-of-the-art prediction algorithms, including PolyPhen-2, SIFT, Mutation Taster, and REVEL. Missense variants will be excluded if: 1) more than two of the aforementioned algorithms predicted the variant to be benign, tolerated, or neutral in effect; and 2) the variant was classified as benign, likely benign, of uncertain significance, or those not reported in ClinVar. *Indicated mutations that exhibited unknown pathogenicity, and were consequently excluded from the analysis.

**Table S3** Detail information of extracted data. The references were presented using PMID, DOI or the article title. Abbreviations: y, years; m, months; d, days; NA, not available; nNO, nasal nitric oxide; FEV1%, forced expiratory volume in 1 second in predicted; FVC%, forced vital capacity in predicted; FEV1/FVC%, forced expiratory volume in 1 second / forced vital capacity in predicted; IDA, inner dynein arm; ODA, outer dynein arm; TEM, transmission electron microscopy.

**Table S4** JBI quality appraisal [checklists](https://www.sciencedirect.com/topics/nursing-and-health-professions/checklist) for case-report, case series and diagnostic test accuracy studies. The overall risk of bias was categorized as low, moderate, or high, based on the confidence ascribed to the study's estimates. The JBI checklists define the study category and utilize eight items, each scored from zero to one, resulting in a final percentage mean. Studies were classified as high quality (>75%), moderate quality (50–74%), or low quality (<50%). Low quality studies were excluded from further analysis and discussion.

**Table S5** Clinical characteristics of PCD patients with variants in *DNAH5*, with an adjusted cut-off age of 18 years at diagnosis.

Data expressed as ^*^median (interquartile range) or ^§^n/N (percentage). N is the total number of patients with available data. Pediatric participants were diagnosed with the disease at an age ≤ 18 years; Adult participants were diagnosed with the disease at an age > 18 years.

Abbreviations: TEM, transmission electron microscopy; IDA, inner dynein arm; ODA, outer dynein arm; Other defects, defects in radial spoke, central pair, micro-tubular disorganization, nexin bridges or no cilia detectable; Immotile cilia, immotile or loss of ciliary motion; Dyskinetic cilia, cilia with minimal residual, limited range, stiff or dyskinetic movements, or with low ciliary beat frequency (CBF); FEV1%, forced expiratory flow in 1 second in predicted; FVC%, forced vital capacity in predicted. *P* value was calculated from the x^2^, Kruskal-Wallis or Mann-Whitney U test. Statistically significant value (*p* < 0.05) is marked in bold.
