# Supplementary figures and images for: Genetic spectrum and genotype–phenotype correlations in DNAH5-mutated primary ciliary dyskinesia: a systematic review
Source: Orphanet J Rare Dis. 2025 Mar 3;20:97. doi: 10.1186/s13023-025-03596-5 (PMC11874857; doi:10.1186/s13023-025-03596-5)

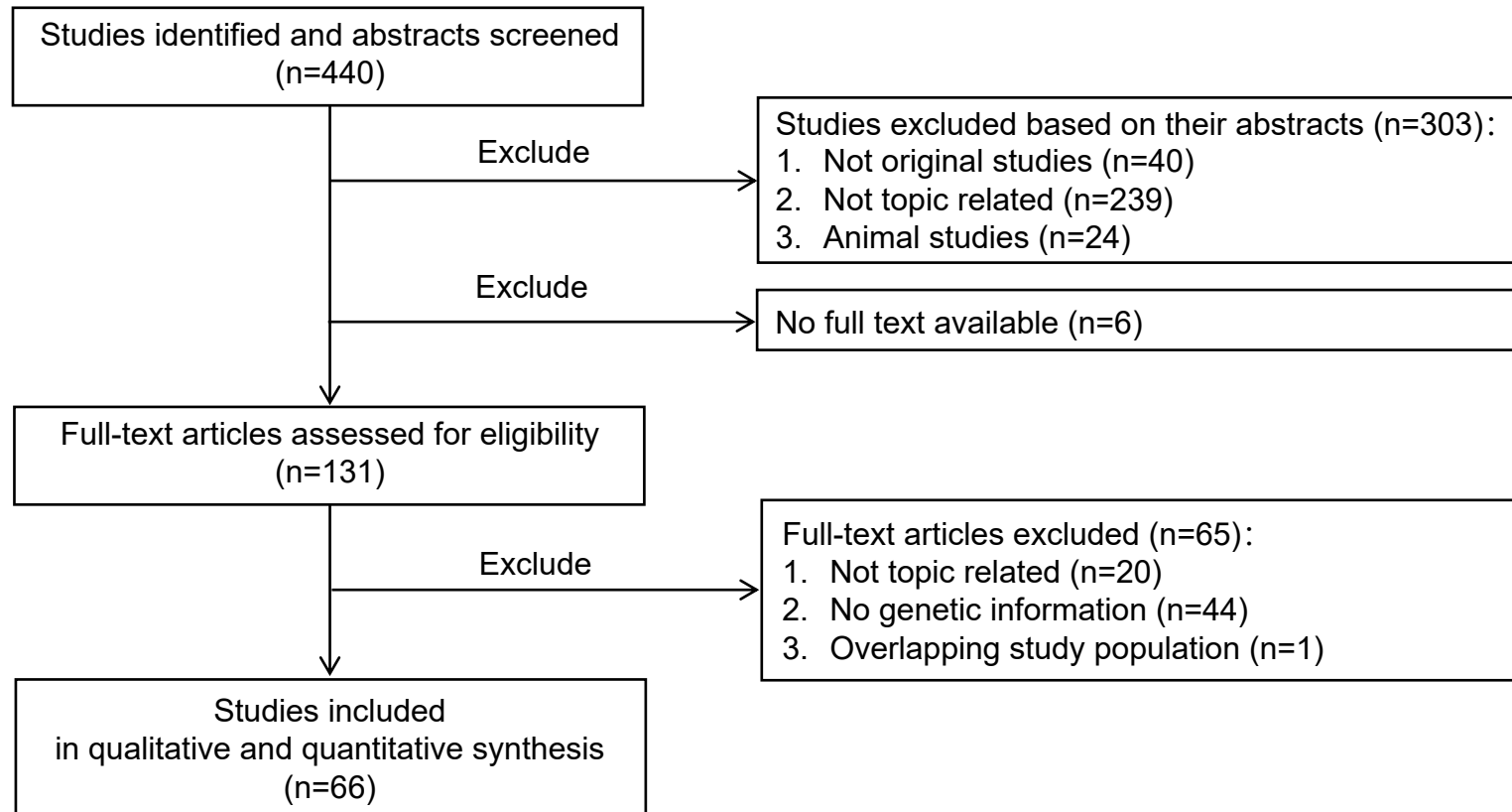

Supplement: Supplementary file 2 — Additional file 2 [file 13023_2025_3596_MOESM2_ESM.pdf]

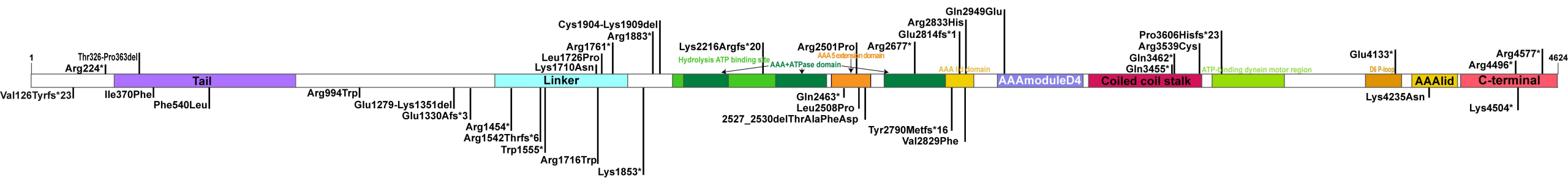

Supplement: Supplementary file 3 — Additional file 3 [file 13023_2025_3596_MOESM3_ESM.pdf]

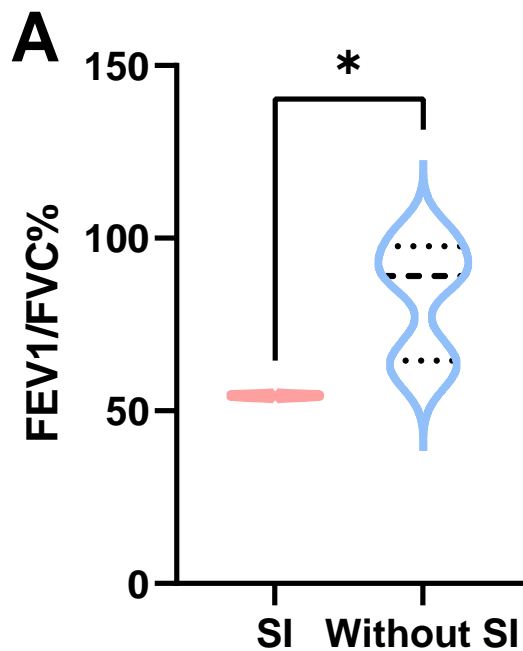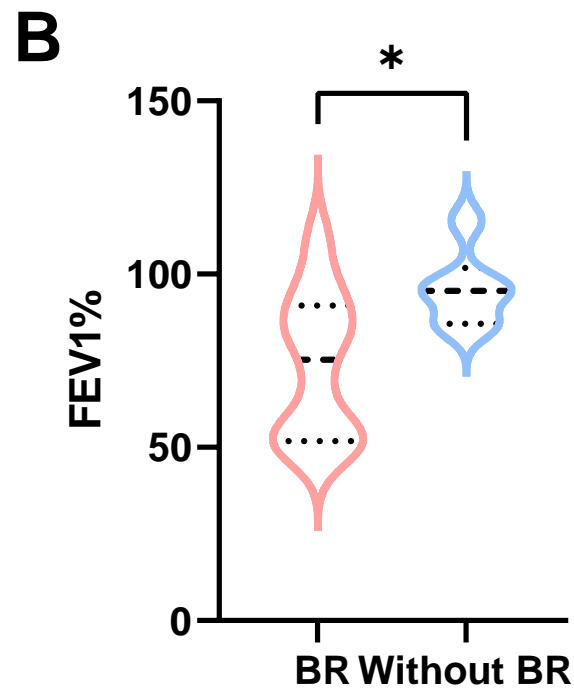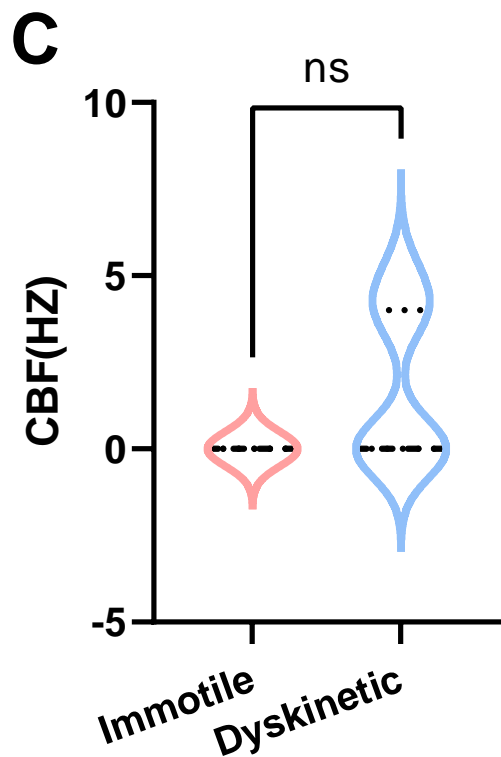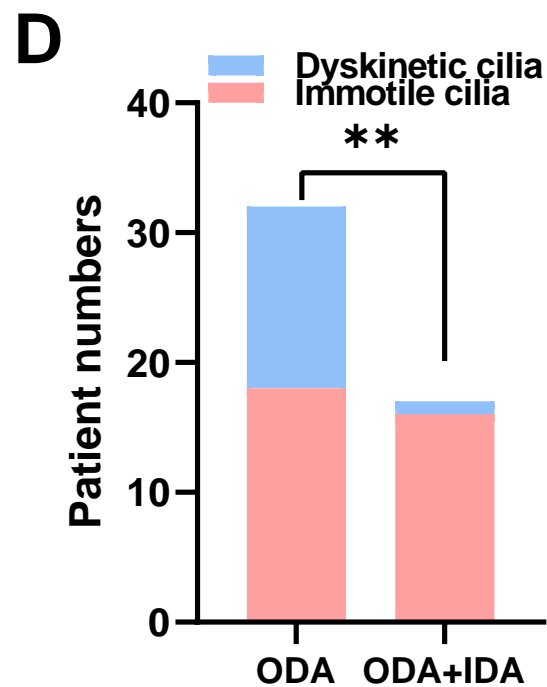

Supplement: Supplementary file 4 — Additional file 4 [file 13023_2025_3596_MOESM4_ESM.pdf]

**A**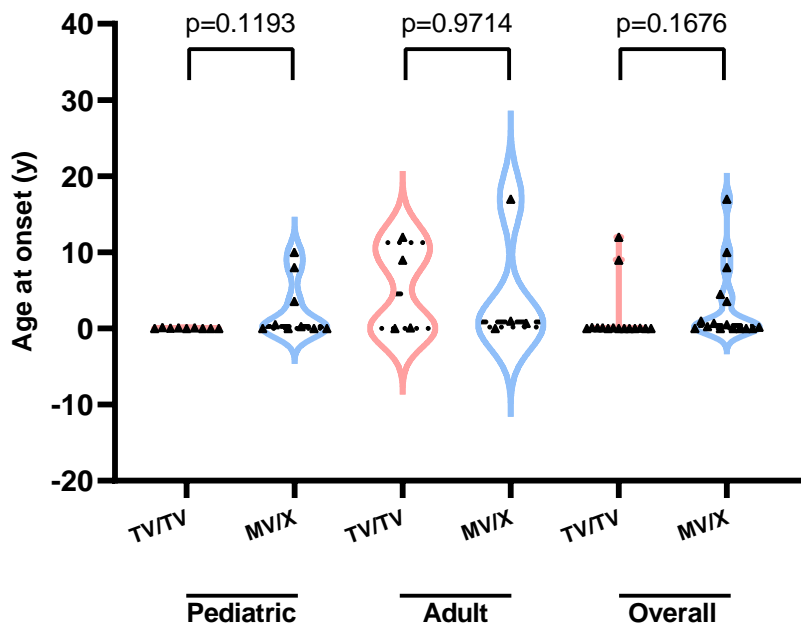**B**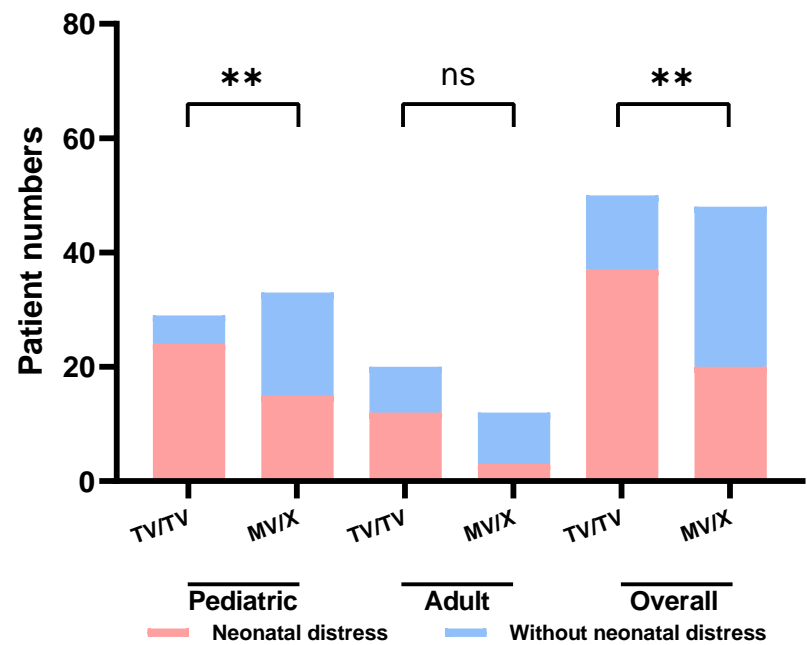**C**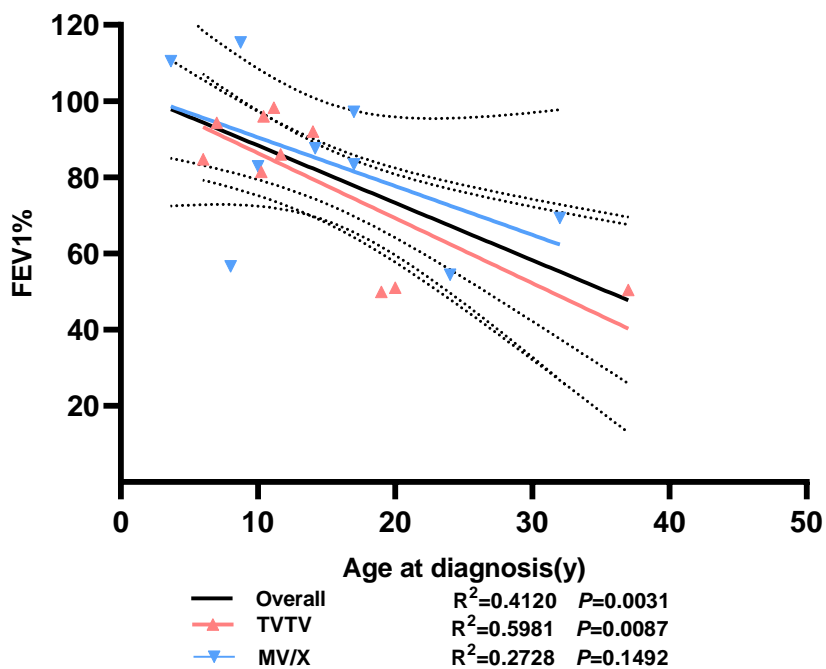**D**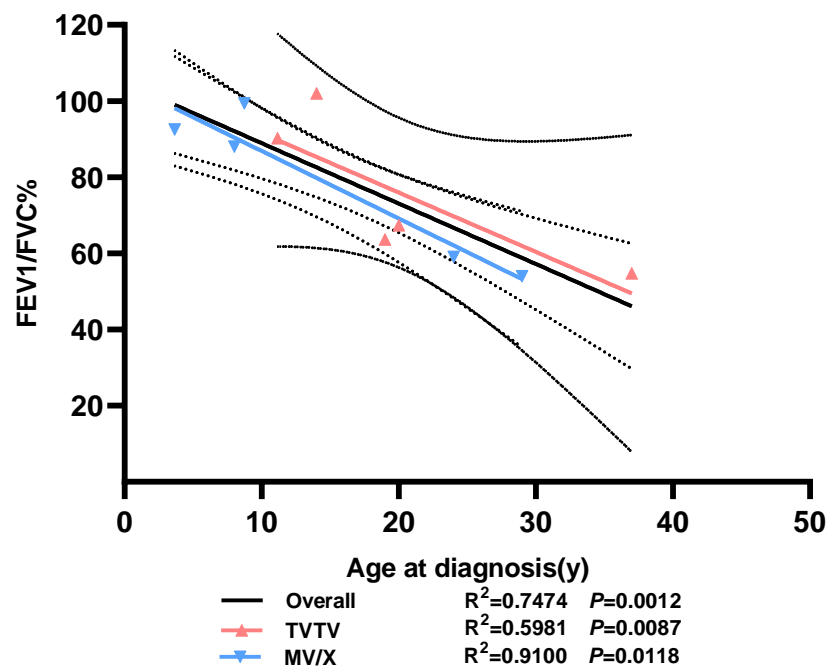

Supplement: Supplementary file 5 — Additional file 5 [file 13023_2025_3596_MOESM5_ESM.pdf]

**A**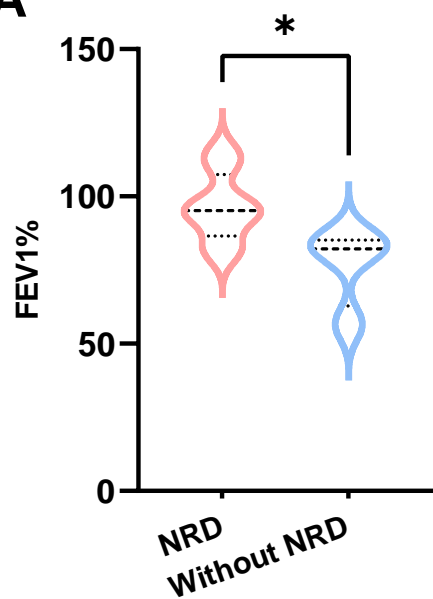**B**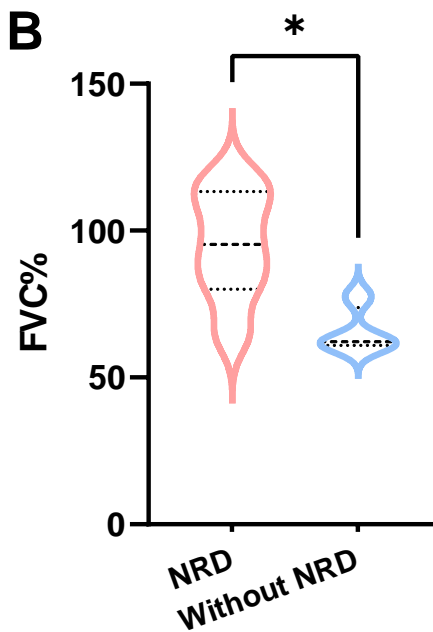**C**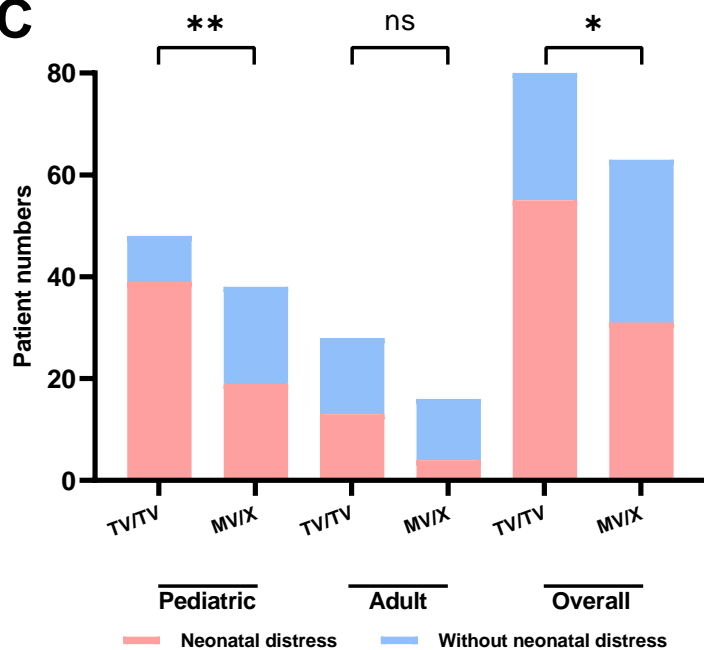**D**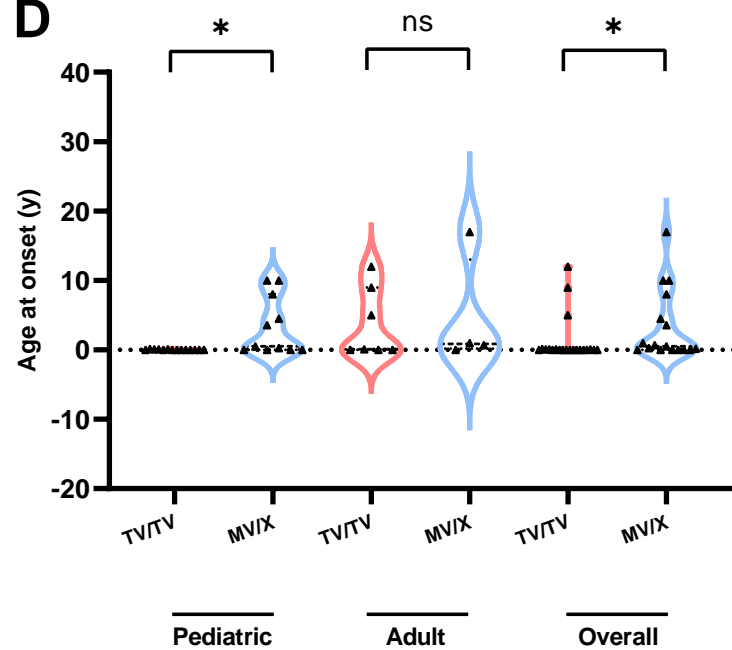

Supplement: Supplementary file 6 — Additional file 6 [file 13023_2025_3596_MOESM6_ESM.pdf]

**A**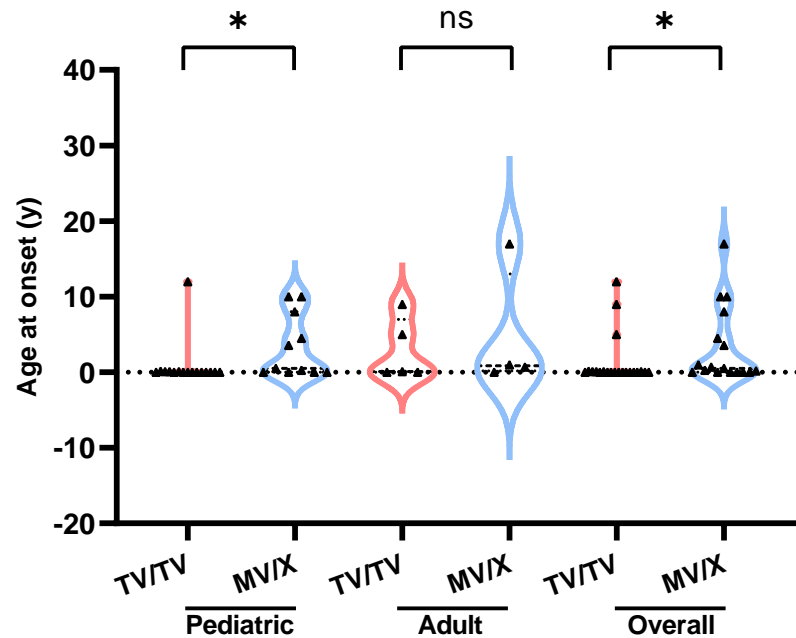**B**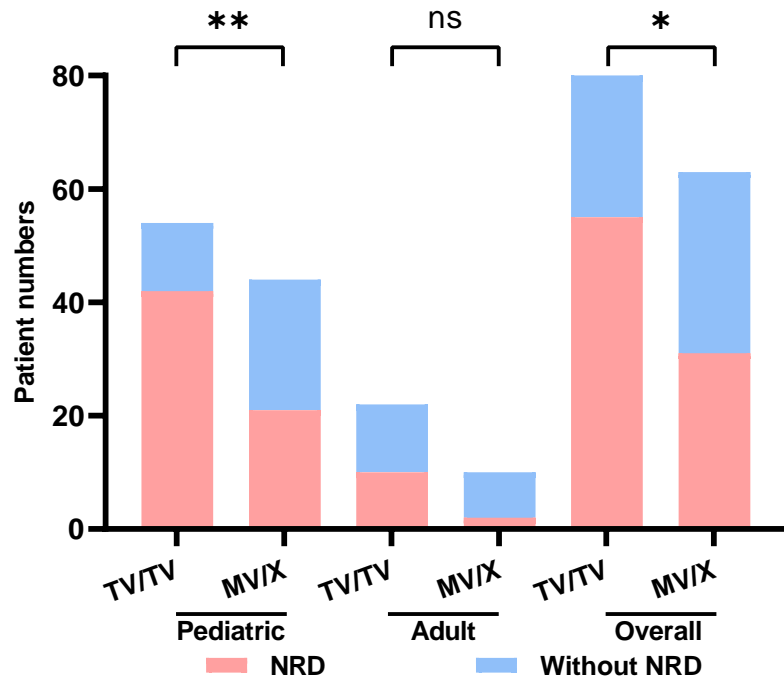

Supplement: Supplementary file 7 — Additional file 7 [file 13023_2025_3596_MOESM7_ESM.pdf]
